# Evaluation of Brain Activity Using Near-infrared Spectroscopy in Inflammatory Bowel Disease Patients

**DOI:** 10.1038/s41598-017-18897-4

**Published:** 2018-01-10

**Authors:** Tatsuo Fujiwara, Soichi Kono, Kyoko Katakura, Kazumichi Abe, Atsushi Takahashi, Naohiko Gunji, Aki Yokokawa, Kazumasa Kawashima, Rieko Suzuki, Akira Wada, Itaru Miura, Hirooki Yabe, Hiromasa Ohira

**Affiliations:** 1 Departments of Gastroenterology, Fukushima Medical University School of Medicine, Fukushima, Japan; 2 Departments of Neuropsychiatry, Fukushima Medical University School of Medicine, Fukushima, Japan; 3 Department of Neuropsychiatry, The University of Tokyo Hospital, Tokyo, Japan

## Abstract

Depression is implicated as a risk factor for the recurrence of inflammatory bowel disease (IBD). Near-infrared spectroscopy (NIRS) and brain-derived neurotrophic factor (BDNF) are useful tools for evaluation of brain activity and a depressive state, respectively. The aim of this study was to clarify the association between brain activity or depressive symptoms and IBD using NIRS and BDNF. This study included 36 ulcerative colitis (UC) patients, 32 Crohn’s disease (CD) patients, and 17 healthy controls (HC). Center for Epidemiologic Studies Depression Scale (CES-D) scores were determined, NIRS was performed, and serum BDNF levels were measured in all subjects. NIRS showed that the mean oxygenated hemoglobin concentration was significantly lower in the frontal lobe in the UC group than in the HC group (HC 167 ± 106 vs. UC 83.1 ± 85.3, p < 0.05). No significant difference was seen between the HC and CD groups. There were also no significant differences in CED-D scores and BDNF levels among the groups. Changes in the NIRS values of the UC group may indicate decreased brain activity and a fundamental difference between UC and CD, which are often lumped together as two types of IBD.

## Introduction

Ulcerative colitis (UC) and Crohn’s disease (CD) are refractory inflammatory bowel diseases (IBD) of uncertain etiology. Psychological factors are thought to be heavily involved in the onset, worsening, and recurrence of IBD^1^. Porcelli and colleagues^1^ found that life events often precede IBD onset or progression. Several studies have shown that anxiety and depression are risk factors for disease exacerbation^2–4^. Moreover, the relationship between psychiatric disorders and the course of IBD has been examined in prospective studies^5–7^. An 18-month prospective study of 60 IBD patients who achieved remission reported that patients with depression on the Beck Depression Inventory (BDI ≥ 13) had a significantly shorter time to recurrence than those without^8^. However, this study did not consider UC and CD separately or evaluate depression in the active phase versus remission.

Several tools are available for diagnosing and evaluating the effects of therapy in psychiatric diseases. Positron emission tomography and functional magnetic resonance imaging are excellent neurological imaging modalities that have advanced our understanding of neurological mechanisms, but the unique imaging environment likely causes subjects’ stress and anxiety, making these modalities of limited utility in assessing psychiatric diseases^9^. Near-infrared spectroscopy (NIRS) is a new modality for evaluating brain function that has a more suitable imaging environment^10,11^. When used to evaluate brain activity, near infrared light readily passes through the body but is absorbed by hemoglobin. This principle is used to estimate changes in oxygenated hemoglobin (oxy-Hb) concentrations in the cerebral cortex. NIRS has many advantages: it is noninvasive and requires only minimal patient restraint; the scanner is compact and portable and therefore usable in many locations; and testing is uncomplicated^12^.

Brain-derived neurotrophic factor (BDNF) is a useful serum marker for evaluation of the pathophysiology of mood disorders, depression, and a range of other psychiatric diseases. On autopsy, levels of messenger RNA and protein levels of BDNF were low in the hippocampus and prefrontal cortex of patients with depression who committed suicide^13^. Moreover, serum BDNF levels decrease in patients with active depression or increasing psychological stress^14–16^, and levels recover following treatment with antidepressant therapy^17^.

No studies have investigated the association between IBD and brain activity using quantitative procedures such as NIRS or serum BDNF levels. Thus, the present study aimed to investigate brain activity and depressive symptoms in patients with UC and CD using NIRS and BDNF and to clarify the correlations between disease activity and brain activity and depressive symptoms.

## Results

### Clinical background characteristics of patients

The clinical background characteristics of the study patients are shown in Table 1. Patients with UC were significantly older than those in the healthy control (HC) group (HC 23 (22 to 65 years) vs. UC 46 (24 to 77 years), p < 0.01). The ages of the patients in the CD and HC groups did not differ significantly (HC 23 (22 to 65 years) vs. CD 23.5 (15 to 52 years), p = 0.34). The sex of the patients in the UC, CD, and HC groups did not differ significantly. A total of 35 patients had total UC, and one patient had UC proctitis. The mean disease duration was 89.4 months. Of the CD patients, one had the ileitis subtype, 29 had the ileocolitis subtype, and two had the colitis subtype. The mean disease duration was 69.4 months. Albumin and C-reactive protein (CRP) levels did not differ significantly between the UC and CD groups.

**Table 1:** Characteristics of the UC, CD, and HC groups.

	UC (n = 36)	CD (n = 32)	Control (n = 17)	p-value
Age(year)	46 (24–77)	23.5 (15–52)	23 (22–65)	<0.01^*^/0.34^**^
Gender(men/women)	19/17	26/6	13/4	0.10^*^/0.61^**^
type	total 35	lleitis 1	—	—
	proctitis 1	lleocolitis 29		
		colitis 2		
Disease duration	89.4 (1–438)	69.4 (1–419)	—	—
active stage	50.5 (2–168)	31.8 (1–95)		
remission stage	97.2 (1–438)	76.3 (2–419)		
Medication				
No treatment	2	0		
5-ASA	15	3		
5-ASA + AZA	4	0		
5-ASA + antiTNF	7	29		
*α* agent				
5-ASA + Steroid	8	0		
Alb(g/dl)	4.2(2.7–5)	4.1(2.5–5)	—	0.14^***^
CRP(mg/dl)	0.11(0.03–16)	0.27(0.03–7)	—	0.37^***^

Data are means and medians (interquartile range). Statistical analysis was conducted using the Mann-Whitney *U* test.

*Control vs. UC. **Control vs. CD. ***UC vs. CD

5-ASA, 5-aminosalicylic acid; AZA, azathioprine;

Anti-TNF-α agent, anti-tumor necrosis factor-α agent.

Alb, Albumin; CRP, C-reactive protein.

### Comparison of the Center for Epidemiologic Studies Depression Scale (CES-D) scores among the UC, CD, and HC groups

**Figure 1:**
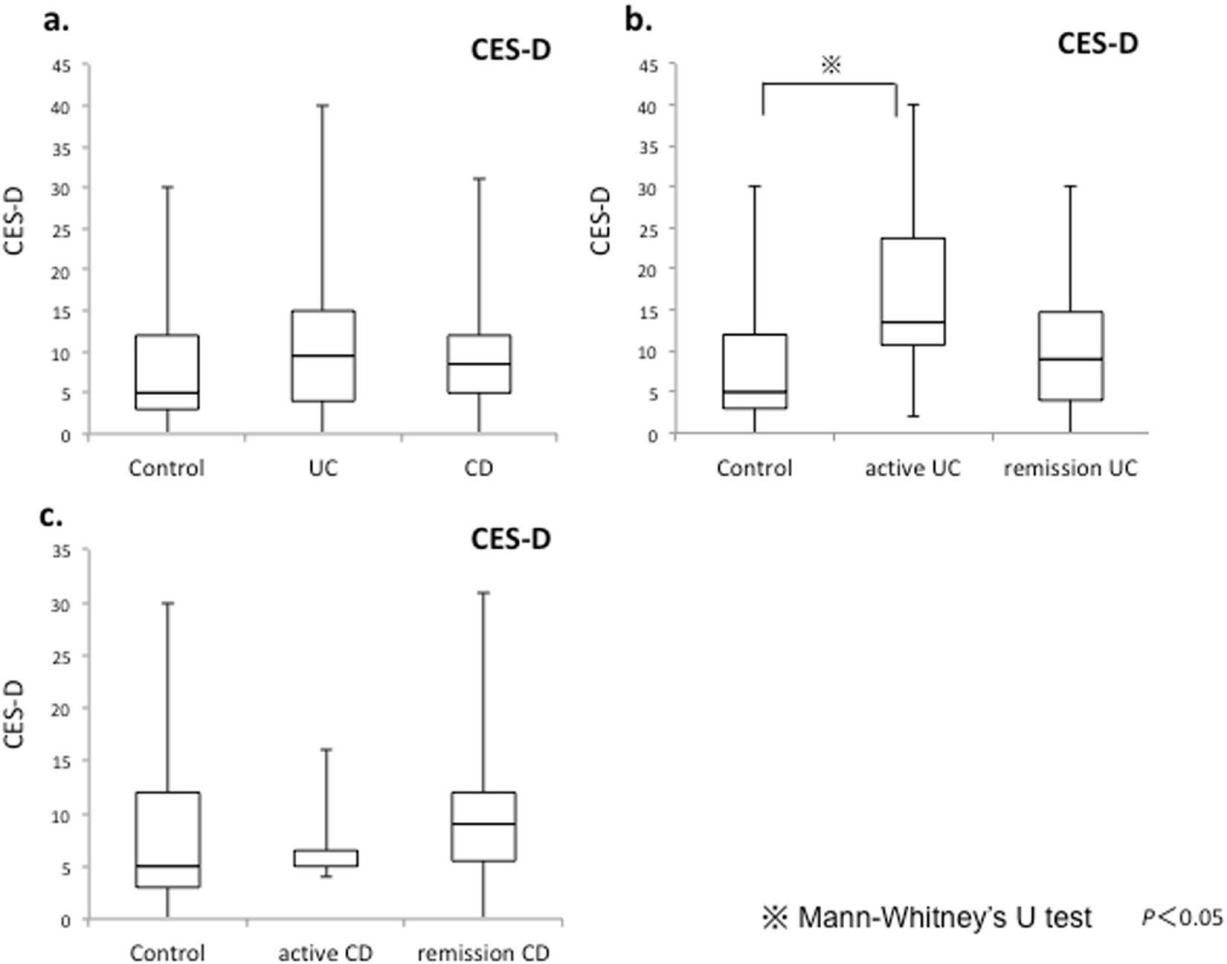
Comparisons of CES-D scores among the HC, UC, and CD groups; HC, active UC, and UC in remission groups; and HC, active CD, and CD in remission groups. (**a**) Scores in the UC and CD groups do not differ significantly from HC group scores. (**b**) Scores in the active UC group are significantly higher compared with the HC group. (**c**) Scores in the active CD and remission CD groups do not differ significantly from HC group scores.

CES-D scores did not differ significantly among the HC, UC, and CD groups (HC 5 (0 to 30) vs. UC 9.5 (0 to 40) vs. CD 8.5 (0 to 31), not significantly different (n.s.)). Comparison of the HC group to the patients with active UC and remission UC showed significantly higher scores in the patients with active UC compared with the HC group (HC 5 (0 to 30) vs. active UC 13.5 (2 to 40), p < 0.05). Scores did not differ significantly among the HC group, patients with active CD, and patients with remission CD (HC 5 (0 to 30) vs. active CD 5 (5 to 16) vs. remission CD 9 (0 to 31), n.s.) (Fig. 1). The ratio of CES-D > 15 was 10/36 (28%) in the UC, 7/32 (22%) in the CD, and 3/17(18%) in the HC groups. No significant difference was seen between the HC group and either the UC or the CD group. In this study, the UC group was significantly older than the HC group (Table 1). To adjust for the effect of age, multiple regression analysis was performed by entering CES-D as a dependent variable and the presence or absence of UC and age as independent variables (n = 53). After adjusting for age, there was a significant association between UC and CES-D (Table S1).

### NIRS data

**Figure 2:**
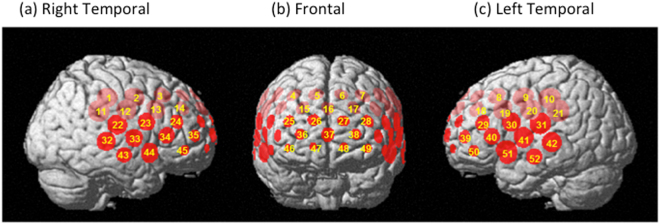
Measurement points of 52 channels for near-infrared spectroscopy. The measuring positions of the device are superimposed on the 3D-reconstructed cerebral surface, based on magnetic resonance imaging, in the right temporal (**a**), frontal (**b**), and left temporal (**c**) brain regions. The 52 measuring positions are labeled channel (ch) 1 to 52, from the right posterior to the left posterior.

As shown in Fig. 2, NIRS is measured by attaching 52 channels to the head. The channels that measure Right Temporal are CHs 1 to 3, 11 to 14, 22 to 24, 32 to 35, and 43 to 45 (Fig. 2a), Front Temporal are CHs 4 to 7, 15 to 17, 25 to 28, 36 to 38, and 46 to 49 (Fig. 2b), and Left Temporal are CHs 8 to 10, 18 to 21, 29 to 31, 39 to 42, and 50 to 52 (Fig. 2c).

**Figure 3:**
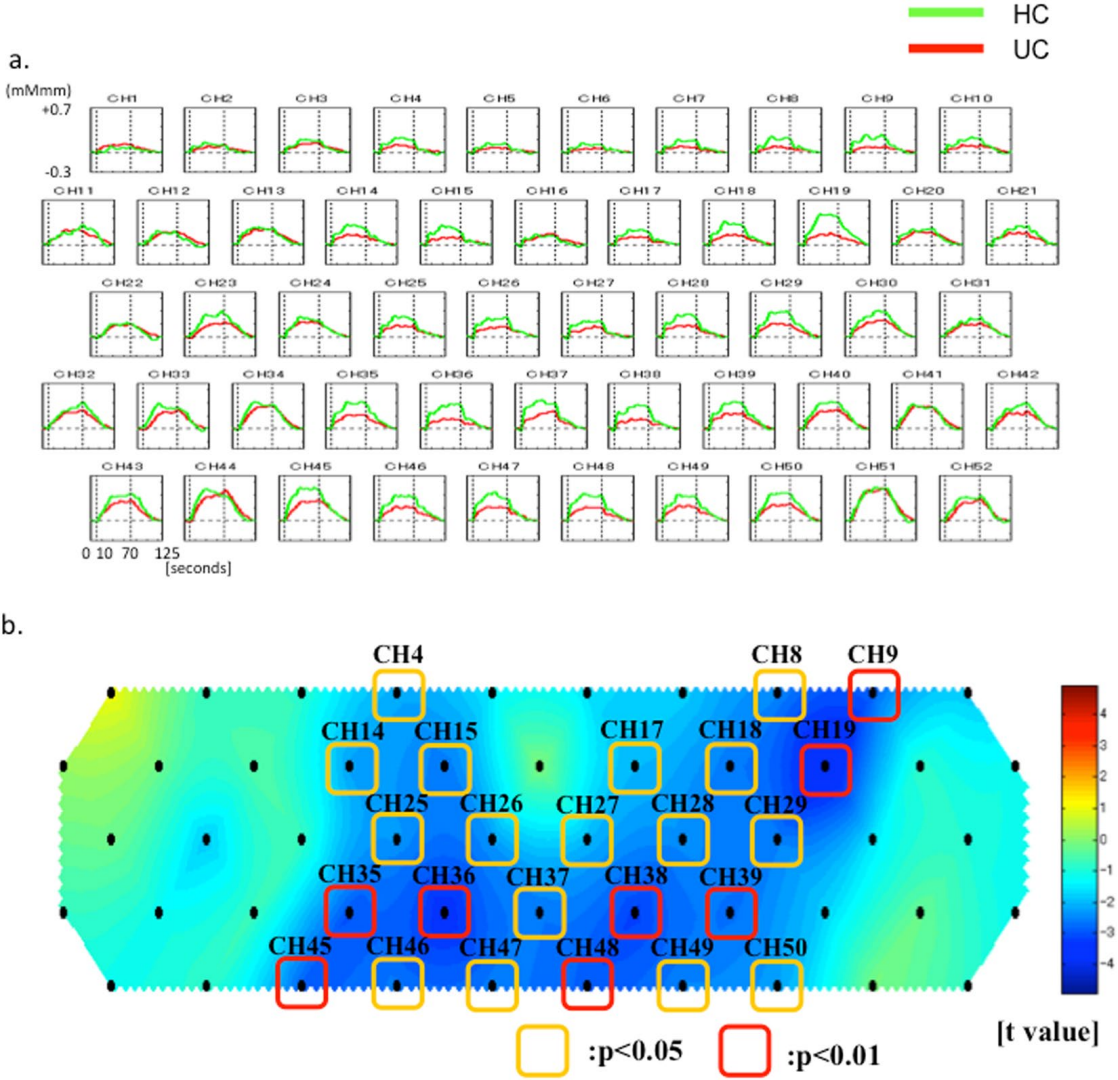
(**a**) Comparison of changes in mean oxy-Hb concentration in the HC (green) and UC (red) groups. (**b**) Topographic map of the differences in mean oxy-Hb concentration changes between the UC and HC groups. THE mean oxy-Hb concentration is significantly decreased in the UC group (p < 0.05 in channels 4, 8, 14, 15, 17, 18, 25 to 29, 37, 46, 47, 49, and 50 (yellow) and p < 0.01 in channels 9, 19, 35, 36, 38, 39, 45, and 48 (red)).

Changes in mean oxy-Hb concentrations in the HC (green) and UC (red) groups are shown in Fig. 3a. The horizontal axis represents time, and the vertical axis represents changes in mean oxy-Hb concentrations (m(mol/l)/mm; mMmm). Figure 3b shows a topographic map of the differences in mean oxy-Hb concentrations. The red, green, and blue colors indicate increases, no change, and decreases of mean oxy-Hb concentrations. The channels with significant differences in mean oxy-Hb concentrations between the HC and UC groups are shown in Fig. 3b. The channels marked with yellow (channels 4, 8, 14, 15, 17, 18, 25 to 29, 37, 46, 47, 49, and 50) were significantly different (p < 0.05), and those marked with red (channels 9, 19, 35, 36, 38, 39, 45, and 48) were also significantly different (p < 0.01) (Table S2). To adjust for the effect of age, multiple regression analysis was performed by entering mean oxy-Hb concentration as a dependent variable and the presence or absence of UC and age as independent variables (n = 53). After adjusting for age, there was an association with borderline significance between UC and NIRS (Table S1).

**Figure 4:**
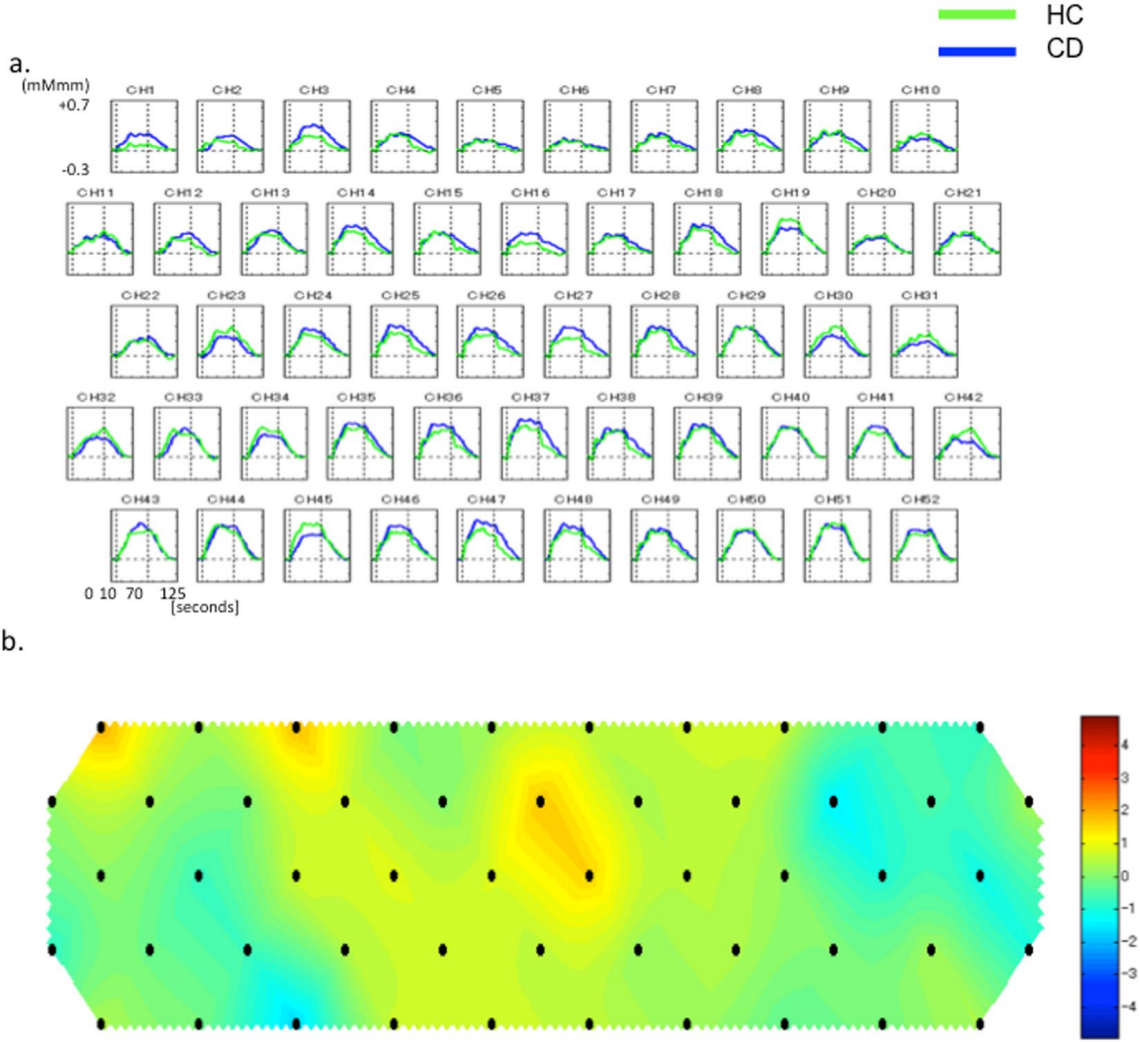
(**a**) Comparison of changes in mean oxy-Hb concentrations in the HC (green) and CD (blue) groups. (**b**) Topographic map of the differences in mean oxy-Hb concentration changes between the CD and HC groups. No significant differences are noted in any channel.

Changes in mean oxy-Hb concentrations in the HC (green) and CD (blue) groups are shown in Fig. 4a. A topographic map with significant differences in mean oxy-Hb concentrations between the HC and CD groups is shown in Fig. 4b. No significant difference was noted in any channel (Table S3).

**Figure 5:**
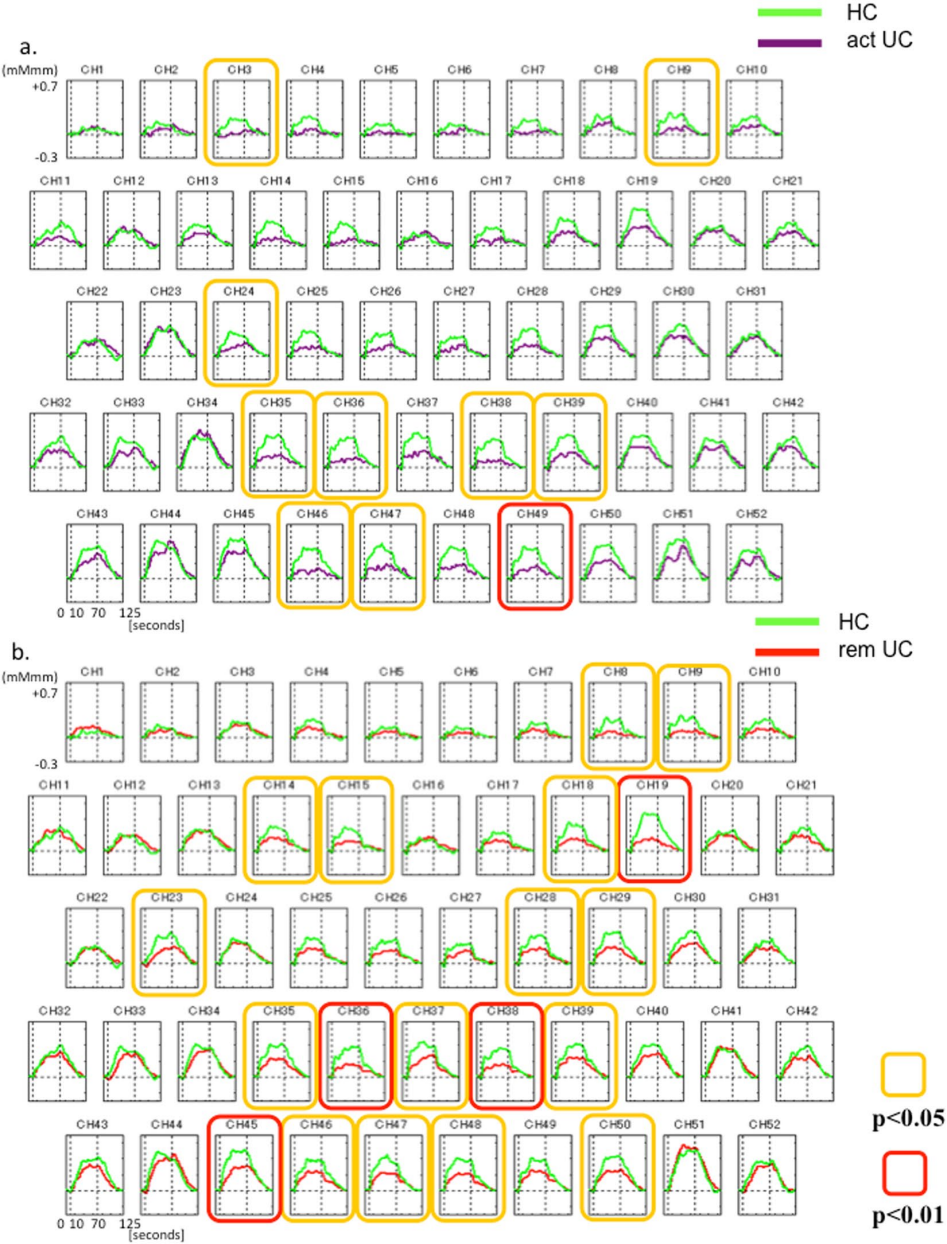
(**a**) Comparison of changes in the mean oxy-Hb concentration in the HC (green) and active UC (purple) groups. The mean oxy-Hb concentration is significantly decreased in the active UC patients (p < 0.05 in channels 3, 9, 24, 35, 36, 38, 39, 46 and 47 (yellow) and p < 0.01 in channel 49 (red)). (**b**) Comparison of changes in the mean oxy-Hb concentration in the HC (green) and UC in remission (red) groups. The mean oxy-Hb concentration is significantly decreased in the patients with UC in remission (p < 0.05 in channels 8, 9, 14, 15, 18, 23, 28, 29, 35, 37, 39, 46 to 48, and 50 (yellow) and p < 0.01 in channels 19, 36, 38, and 45 (red)).

Changes in mean oxy-Hb concentrations in the HC (green) and active UC (purple) groups are shown in Fig. 5a. Mean oxy-Hb concentrations were significantly decreased in the active UC patients (p < 0.05 in channels 3, 9, 24, 35, 36, 38, 39, 46 and 47 (yellow) and p < 0.01 in channel 49 (red)) (Table S4).

Changes in mean oxy-Hb concentrations in the HC (green) and UC in remission (red) groups are shown in Fig. 5b. Mean oxy-Hb concentrations were significantly decreased in the patients with UC in remission (p < 0.05 in channels 8, 9, 14, 15, 18, 23, 28, 29, 35, 37, 39, 46 to 48, and 50 (yellow) and p < 0.01 in channels 19, 36, 38, and 45 (red)) (Table S5).

Changes in mean oxy-Hb concentrations did not differ significantly between the patients with active UC and UC in remission. This was also true for the patients with CD.

Integral values of mean oxy-Hb concentrations in the frontal lobe were compared among the groups. The mean oxy-Hb concentration was significantly decreased in the UC group compared with the HC group (HC 167 ± 106 vs. UC 83.1 ± 85.3, p < 0.05). On the other hand, the mean oxy-Hb concentration did not differ significantly between the HC and CD groups (HC 167 ± 106 vs. CD 203 ± 176, p = 0.43). Moreover, the mean oxy-Hb concentration was decreased in the patients with active UC or remission UC compared with the HC group (HC 167 ± 106 vs. act UC 58.7 ± 63.5 vs. rem UC 87.9 ± 89.2, p < 0.05). The mean oxy-Hb concentration in the HC group did not differ significantly from that in the patients with active CD or remission CD (HC 167 ± 106 vs. act CD 198 ± 220 vs. rem CD 204 ± 172, n.s.).

The above data show that the mean oxy-Hb concentration was decreased in patients with UC irrespective of disease activity. The data suggest that the mean oxy-Hb concentration was likewise unaffected by disease activity in patients with CD, since the mean oxy-Hb concentrations in the patients with active CD and remission CD did not differ from those in the HC group.

### BDNF data

BDNF levels in the HC, UC, and CD groups were not significantly different (HC 4.71 (3.26 to 6.08) vs. UC 5.18 (2.94 to 6.29) vs. CD 4.98 (3.65 to 6.34) ng/mL, n.s.). Levels likewise did not differ significantly among the HC group, patients with active UC, and remission UC (HC 4.71 (3.26 to 6.08) vs. active UC 4.79 (4.01 to 5.48) vs. remission UC 5.21 (2.51 to 6.29) ng/mL, n.s.) or among the HC group, patients with active CD, and patients with remission CD (HC 4.71 (3.26 to 6.08) vs. active CD 5.16 (3.65 to 6.26) vs. remission CD 4.94 (3.82 to 6.75) ng/mL, n.s.).

## Discussion

This study evaluated brain activity and depressive symptoms in patients with IBD using NIRS and BDNF for the first time. NIRS showed that the mean oxy-Hb concentration was significantly decreased in the UC group compared with the HC group regardless of UC disease activity. On the other hand, the mean oxy-Hb concentration did not differ significantly between the HC and CD groups. In addition, there were no significant differences in the CES-D scores and BDNF levels among the HC, UC, and CD groups.

The relationship between psychiatric factors and IBD has long been a subject of debate and remain to be clearly demonstrated. The difficulty in establishing this relationship is due in part to the wide-ranging effects of psychiatric factors, the sheer number of factors and difficulty testing for them, and the lack of quantitative tests; thus, comparison is difficult. Many reports have shown a relationship between IBD and depression using the self-rating scale. Brain activity and depressive symptoms in patients with IBD were evaluated using CES-D, NIRS, and BDNF levels. The CES-D is a commonly used, self-reported depression scale. BDNF levels have been proposed to quantitatively measure depression. NIRS using near infrared light is a method to estimate and evaluate blood volume changes of the cerebral cortex using the characteristic that near infrared light passes easily through a living body but is easily absorbed by the hemoglobin that is present. NIRS is covered by National Health Insurance in Japan and helps distinguish between healthy persons, schizophrenia, bipolar disorder, and depression. The frontotemporal NIRS signal has been proposed as a supportive tool for assisting with the diagnosis of major psychiatric disorders with depressive symptoms, in addition to evaluation of brain activity^10,11^. A decrease in the oxy-Hb concentration with NIRS reflects a decrease in frontal lobe function in patients with depression or a depressed state^18,19^. We recently reported reduced frontal activation in chronic hepatitis C patients receiving interferon-based therapy^20^ and in female patients with non-alcoholic fatty liver disease using NIRS^21^.

With NIRS, mean oxy-Hb concentrations were lower in the UC group than in the HC group in many of the 52 channels. The decrease in the mean oxy-Hb concentration in the frontal lobe was similar to the results seen in patients with depression^18,19^. However, patients with UC were not evaluated for depression using both CES-D and BDNF levels. Some studies recently reported that depression may be associated with cognitive impairments^22,23^, and early-onset depression may be related to deficits in attention, memory, and executive functioning^24,25^. Another previous study showed a correlation between inflammation and depression^26^. Prospective observational studies have shown that high blood CRP levels increase the risk of depression^27^. These facts may imply that the decreased NIRS signal in UC patients was the result of inflammation. However, NIRS signals were decreased in UC patients with both active disease and remission. Therefore, NIRS in patients with UC may reflect a decrease in overall brain activity.

Some studies have shown a correlation between CD and depression^28,29^. However, all results for CES-D, BDNF levels, and NIRS values in patients with CD were equivalent to those in the HC group in the present study. Although UC and CD are often lumped together as two types of IBD, the differences between the two conditions in the present study may be indicative of a fundamental difference between UC and CD.

There are several limitations to this study. First, the UC group was significantly older than the HC group (Table 1). To examine the effects of age, multiple regression analysis was performed with mean oxy-Hb concentration as a dependent variable and the presence or absence of UC and age as independent variables (n = 53). Even after adjusting for age, there was a borderline significant relationship between UC and NIRS (Table S1). Second, the reproducibility of NIRS and intra-individual variance are issues. There is a possibility that the oxy-Hb concentration changes decrease with sleepiness and anxiety when NIRS measurements are reported^30^. Therefore, in this study, efforts were made to increase the reproducibility of NIRS data by matching the conditions to the extent possible, such as performing NIRS in the morning. Furthermore, to investigate intra-individual variance in an additional study, 10 mentally healthy subjects were measured at intervals of 12 weeks, and the reliability of the NIRS test-retest was assessed. The Intraclass Correlation Coefficient (ICC) of the mean oxy-Hb concentration during the task segment was calculated for the 52 channels. The single-measure ICC was 0.5309, and the average measure ICC was 0.6936, which are both reliable, as described by Kakimoto *et al*.^31^. Finally, it was not possible to examine the results of the active stage and the remission stage in the same patient. There were few cases, 5 UC cases and 3 CD cases, that were assessed in both the active stage and the remission stage, but there were no clear trends in CES-D, NIRS, and BDNF in the cases assessed. A longitudinal study including changes in the active stage and remission stage would be very interesting and could be evaluated by increasing the number of patients in the future.

## Materials and Methods

### Study group

The study group included 68 patients who visited the Department of Gastroenterology of the School of Medicine, Fukushima Medical University on an outpatient basis from October 2015 to October 2016 and consented to participate in this study and 17 volunteers, for a total of 85 participants. The study was approved by the ethics committee of Fukushima Medical University (#2495) and was conducted in compliance with the Declaration of Helsinki. All subjects provided their written, informed consent to participate in the study. Of the 36 patients with UC, six had disease in the active phase, and 30 were in remission. Partial Mayo scores^32^ were used to evaluate disease activity. Remission was defined as a Mayo score of no greater than 2 points and no individual sub-score exceeding 1 point. Of the 32 patients with CD, five had disease in the active phase, and 27 were in remission. The Crohn’s Disease Activity Index (CDAI)^33^ was used to evaluate disease activity. A CDAI score of less than 150 constituted remission. Patients receiving treatment for schizophrenia, depression, or bipolar disorder were excluded.

The CES-D^34^ was used to evaluate depression in each group. Age and sex were compared as patient demographic characteristics. The disease subtype, disease duration, and serum albumin and CRP levels were determined in the patients in the UC and CD groups. All patients underwent NIRS and measurements of BDNF. Serum albumin, CRP, NIRS, and BDNF were analyzed on the same day.

### NIRS measurements

**Figure 6:**
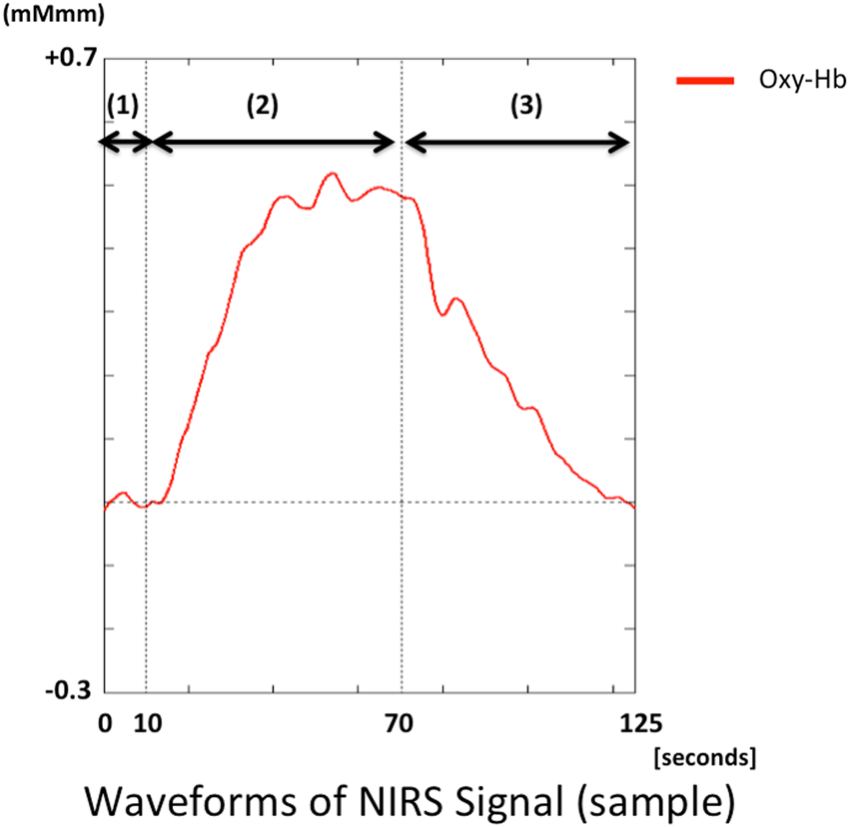
Time-dependent changes in oxy-hemoglobin (oxy-Hb) concentration in an NIRS signal waveform (sample). The average waveforms of time-dependent changes in mean oxy-Hb concentrations in the channels. (1) 10-s pre-task baseline period (2) 60-s activation period (3) in the 55-s post-task baseline period.

NIRS measurements have been discussed previously^11^. In this study, oxy-Hb, deoxy-Hb, and total Hb were measured with a 52-channel NIRS machine (Hitachi ETG400 Hitachi Medical Corp., Tokyo, Japan) using two wavelengths of near-infrared light (695 and 830 nm). A verbal fluency task (VFT) was used as an activating task during NIRS analysis. The 52-channel device was connected symmetrically around the prefrontal cortex^35^ (Fig. 2). The lower and forward-most channels were placed along the line connecting T3-Fpz-T4, based on the international 10–20 system. Compliance with the scalp measurement sites of the international 10–20 system allows prediction of the measurement sites on the brain surface with relatively high accuracy^35,36^. NIRS signal changes were measured during a 10-s pre-task baseline period, a 60-s activation period, and a 55-s post-task baseline period (Fig. 6). The rate of oxy-Hb concentration data sampling was 0.1 s. The obtained data were analyzed using the “integral mode”: the pre-task baseline was set as the mean over a 10-s period just before the task period, and the post-task baseline was fixed as the mean over the last 5-s of the post task period. Linear fitting between the pre and post-task baselines was applied to data between the two baselines. The average oxy-Hb concentration during the VFT that was performed for 60 seconds was used for the analysis. The algorithm developed by Takizawa *et al*.^11^ was used to automatically reject data with artifacts. Data are expressed as waveforms and topographic maps.

### Activation task

The VFT is widely used to test frontal lobe function^9^ and is commonly used with NIRS analysis. Changes in hemoglobin oxygenation occur in people performing the VFT. Artifacts must be eliminated by having the subject sit in a chair, relax, and move as little as possible during the test. The subject is first prompted by a voice saying, “Start /a/, /i/, /u/, /e/, /o/” to repeat the utterance “/a/, /i/, /u/, /e/, /o/” for 30 s. The baseline activity recorded during this meaningless utterance is used to remove the effect of vocalization on brain activity from the data. The subject is next prompted by voice to vocalize as many words as possible that start with a certain letter. This is done in three 20-s sets. The subjects are verbally prompted to vocalize words starting with a certain letter to increase the difficulty of the task. The exercise is scored by recording the number of words uttered every 20 s. Finally, the subject is prompted by a voice saying, “Stop /a/, /i/, /u/, /e/, /o/” to stop the task and repeat “/a/, /i/, /u/, /e/, /o/” for 70 s.

### Questionnaire

All participants were assessed with the CES-D before undergoing NIRS analysis. Widely used to help inform the diagnosis of depression, the CES-D is a self-administered instrument^34^ with the following assessment criteria: <15, no depression; 15–21, mild to moderate depression; and >21, possibility of major depression. A positive score of 16 or more indicates the possibility of depression.

### BDNF assay

Serum BDNF levels were determined with the BDNF Emax ImmunoAssay Kit (Promega, Madison, WI). A carbonate buffer (pH 9.7) was prepared on the day before analysis and used to dilute the anti-BDNF monoclonal antibody reagent by a factor of 1000. The resulting solution was pipetted into an ELISA plate at 100 μL per well and allowed to stand for 18 h at 4 °C. The wells were rinsed once with TBS-T, which was replaced with blocking buffer, and allowed to stand at room temperature for 1 h. The wells were then rinsed once with TBS-T, which was replaced with the samples, and the plate was shaken on a shaker for 2 h. The wells were then rinsed five times with TBS-T, which was replaced with anti-human BDNF polyclonal antibody reagent. The plate was returned to the shaker and shaken for 2 h. The wells were rinsed five times with TBS-T, which was replaced with anti-IgY HRP, and the plate was shaken on a shaker for 1 h. The wells were rinsed five times with TBS-T, and TMB One Solution was added. The plate was shaken for 10 min until the solution turned blue, and then the reaction was stopped with 1 N hydrochloric acid. Absorbance was determined at 450 nm using the xMark system (Bio-Rad). The range of the standard curve was 7.8 to 500 pg/mL.

### Statistical analysis

Continuous data are expressed as medians (interquartile range). The three groups (HC vs. UC vs. CD) were compared using the Mann-Whitney *U* test. For the analysis of NIRS data, the averages of oxy-Hb, deoxy-Hb, and total-Hb were calculated and analyzed using one-way repeated-measures ANOVA. All statistical analyses were carried out using SPSS 24.0 J software (IBM Inc., Armonk, NY, USA) and MATLAB R2011 (MathWorks Inc., Natick, MA). A p value < 0.05 was considered significant.

## Conclusions

The quantitative data produced from the NIRS and BDNF tests in this study allow comparisons among patients with different disease variants and activity levels. We hope that the present study helps further efforts to establish a clear correlation between IBD and psychiatric factors.

## Electronic supplementary material

Supplementary Information

## References

[1] Porcelli P, Zaka S, Tarantino S, Sisto G (1993). [Body Image Index in the Rorschach test in ulcerative proctocolitis]. Minerva Psichiatr.

[2] Calvet X (2006). Remission on thiopurinic immunomodulators normalizes quality of life and psychological status in patients with Crohn’s disease. Inflamm Bowel Dis.

[3] Levenstein S (1994). Psychological stress and disease activity in ulcerative colitis: a multidimensional cross-sectional study. Am J Gastroenterol.

[4] Maunder RG (2006). Psychobiological subtypes of ulcerative colitis: pANCA status moderates the relationship between disease activity and psychological distress. Am J Gastroenterol.

[5] Porcelli P, Leoci C, Guerra V (1996). A prospective study of the relationship between disease activity and psychologic distress in patients with inflammatory bowel disease. Scand J Gastroenterol.

[6] Mardini HE, Kip KE, Wilson JW (2004). Crohn’s disease: a two-year prospective study of the association between psychological distress and disease activity. Dig Dis Sci.

[7] Mikocka-Walus AA (2008). Does psychological status influence clinical outcomes in patients with inflammatory bowel disease (IBD) and other chronic gastroenterological diseases: an observational cohort prospective study. Biopsychosoc Med.

[8] Mittermaier C (2004). Impact of depressive mood on relapse in patients with inflammatory bowel disease: a prospective 18-month follow-up study. Psychosom Med.

[9] Klumpp H, Deldin P (2010). Review of brain functioning in depression for semantic processing and verbal fluency. Int J Psychophysiol.

[10] Suto T, Fukuda M, Ito M, Uehara T, Mikuni M (2004). Multichannel near-infrared spectroscopy in depression and schizophrenia: cognitive brain activation study. Biol Psychiatry.

[11] Takizawa R (2008). Reduced frontopolar activation during verbal fluency task in schizophrenia: a multi-channel near-infrared spectroscopy study. Schizophr Res.

[12] Irani F, Platek SM, Bunce S, Ruocco AC, Chute D (2007). Functional near infrared spectroscopy (fNIRS): an emerging neuroimaging technology with important applications for the study of brain disorders. Clin Neuropsychol.

[13] Dwivedi Y (2003). Altered gene expression of brain-derived neurotrophic factor and receptor tyrosine kinase B in postmortem brain of suicide subjects. Arch Gen Psychiatry.

[14] Brunoni AR, Lopes M, Fregni F (2008). A systematic review and meta-analysis of clinical studies on major depression and BDNF levels: implications for the role of neuroplasticity in depression. Int J Neuropsychopharmacol.

[15] Bocchio-Chiavetto L (2010). Serum and plasma BDNF levels in major depression: a replication study and meta-analyses. World J Biol Psychiatry.

[16] Sen S, Duman R, Sanacora G (2008). Serum brain-derived neurotrophic factor, depression, and antidepressant medications: meta-analyses and implications. Biol Psychiatry.

[17] Mitoma M (2008). Stress at work alters serum brain-derived neurotrophic factor (BDNF) levels and plasma 3-methoxy-4-hydroxyphenylglycol (MHPG) levels in healthy volunteers: BDNF and MHPG as possible biological markers of mental stress?. Prog Neuropsychopharmacol Biol Psychiatry.

[18] Matsuo K, Kato T, Fukuda M, Kato N (2000). Alteration of hemoglobin oxygenation in the frontal region in elderly depressed patients as measured by near-infrared spectroscopy. J Neuropsychiatry Clin Neurosci.

[19] Matsuo K, Kato N, Kato T (2002). Decreased cerebral haemodynamic response to cognitive and physiological tasks in mood disorders as shown by near-infrared spectroscopy. Psychol Med.

[20] Abe K (2017). Reduced frontal activation during verbal fluency task in chronic hepatitis C patients with interferon-based therapy as measured by near-infrared spectroscopy. Hepatol Res.

[21] Takahashi A (2017). Reduced brain activity in female patients with non-alcoholic fatty liver disease as measured by near-infrared spectroscopy. PLoS One.

[22] Castaneda AE, Tuulio-Henriksson A, Marttunen M, Suvisaari J, Lonnqvist J (2008). A review on cognitive impairments in depressive and anxiety disorders with a focus on young adults. J Affect Disord.

[23] Gualtieri CT, Johnson LG, Benedict KB (2006). Neurocognition in depression: patients on and off medication versus healthy comparison subjects. J Neuropsychiatry Clin Neurosci.

[24] Castaneda AE (2008). Cognitive functioning in a population-based sample of young adults with a history of non-psychotic unipolar depressive disorders without psychiatric comorbidity. J Affect Disord.

[25] Gunther T, Holtkamp K, Jolles J, Herpertz-Dahlmann B, Konrad K (2004). Verbal memory and aspects of attentional control in children and adolescents with anxiety disorders or depressive disorders. J Affect Disord.

[26] Thaler JP (2012). Obesity is associated with hypothalamic injury in rodents and humans. J Clin Invest.

[27] Daly M (2013). The relationship of C-reactive protein to obesity-related depressive symptoms: a longitudinal study. Obesity (Silver Spring).

[28] Paulley JW (1974). Psychological management of Crohn’s disease. Practitioner.

[29] Mawdsley JE, Rampton DS (2005). Psychological stress in IBD: new insights into pathogenic and therapeutic implications. Gut.

[30] Suda M (2008). Decreased cortical reactivity underlies subjective daytime light sleepiness in healthy subjects: a multichannel near-infrared spectroscopy study. Neurosci Res.

[31] Kakimoto Y (2009). Intrasubject reproducibility of prefrontal cortex activities during a verbal fluency task over two repeated sessions using multi-channel near-infrared spectroscopy. Psychiatry Clin Neurosci.

[32] Schroeder KW, Tremaine WJ, Ilstrup DM (1987). Coated oral 5-aminosalicylic acid therapy for mildly to moderately active ulcerative colitis. A randomized study. N Engl J Med.

[33] Best WR, Becktel JM, Singleton JW (1979). Rederived values of the eight coefficients of the Crohn’s Disease Activity Index (CDAI). Gastroenterology.

[34] Weissman MM, Sholomskas D, Pottenger M, Prusoff BA, Locke BZ (1977). Assessing depressive symptoms in five psychiatric populations: a validation study. Am J Epidemiol.

[35] Tsuzuki D (2007). Virtual spatial registration of stand-alone fNIRS data to MNI space. Neuroimage.

[36] Okamoto M (2004). Three-dimensional probabilistic anatomical cranio-cerebral correlation via the international 10–20 system oriented for transcranial functional brain mapping. Neuroimage.

