# Supplementary material for: Evaluation of Brain Activity Using Near-infrared Spectroscopy in Inflammatory Bowel Disease Patients
Source: Sci Rep. 2018 Jan 10;8:402. doi: 10.1038/s41598-017-18897-4 (PMC5762642; doi:10.1038/s41598-017-18897-4)
Supplement: Supplementary file 1 — Supplementary Information [file 41598_2017_18897_MOESM1_ESM.pdf]

Supplementary Information

Evaluation of Brain Activity Using Near-infrared Spectroscopy in Inflammatory Bowel Disease Patients

Tatsuo Fujiwara*[1], Soichi Kono[2], Kyoko Katakura[1], Kazumichi Abe[1], Atsushi Takahashi[1], Naohiko Gunji[1], Aki Yokokawa[1], Kazumasa Kawashima[1], Rieko Suzuki[2], Akira Wada[2,3], Itaru Miura[2], Hirooki Yabe[2] and Hiromasa Ohira[1]

Departments of [1]Gastroenterology, and [2]Neuropsychiatry, Fukushima Medical University School of Medicine, Fukushima, Japan. [3]Department of Neuropsychiatry, The University of Tokyo Hospital, Tokyo, Japan.

Table S1. Multiple regression model included CES-D or NIRS as dependent variable and the age and UC as independent variables.

	Variable	β	p-value
CES-D	Age, per 1 year	−0.26	0.126
	UC, yes	0.33	0.049
NIRS	Age, per 1 year	−0.15	0.349
	UC, yes	−0.31	0.053

CES-D, Center for Epidemiologic Studies Depression Scale;

NIRS, Near-infrared spectroscopy;

β, standardized coefficients beta;

Table S2. Mean and standard deviation for each channel of HC and UC

	HC	UC	p-value
CH1	0.05 ± 0.15	0.10 ± 0.14	0.290
CH2	0.10 ± 0.18	0.07 ± 0.16	0.433
CH3	0.14 ± 0.13	0.10 ± 0.16	0.407
CH4	0.17 ± 0.16	0.08 ± 0.11	0.040*
CH5	0.10 ± 0.17	0.05 ± 0.07	0.147
CH6	0.09 ± 0.12	0.04 ± 0.10	0.116
CH7	0.14 ± 0.12	0.07 ± 0.13	0.084
CH8	0.16 ± 0.13	0.06 ± 0.14	0.018*
CH9	0.20 ± 0.13	0.06 ± 0.14	0.008*
CH10	0.16 ± 0.16	0.08 ± 0.12	0.070
CH11	0.15 ± 0.19	0.16 ± 0.15	0.856
CH12	0.14 ± 0.22	0.13 ± 0.13	0.830
CH13	0.21 ± 0.17	0.18 ± 0.19	0.649
CH14	0.23 ± 0.19	0.12 ± 0.15	0.029*
CH15	0.21 ± 0.25	0.08 ± 0.11	0.011*
CH16	0.10 ± 0.18	0.11 ± 0.12	0.998
CH17	0.17 ± 0.16	0.09 ± 0.12	0.048*
CH18	0.25 ± 0.17	0.11 ± 0.18	0.011*
CH19	0.35 ± 0.21	0.12 ± 0.18	<0.001*
CH20	0.18 ± 0.20	0.14 ± 0.12	0.416
CH21	0.19 ± 0.07	0.14 ± 0.14	0.254
CH22	0.14 ± 0.17	0.12 ± 0.16	0.825
CH23	0.25 ± 0.15	0.13 ± 0.17	0.072
CH24	0.22 ± 0.11	0.18 ± 0.19	0.480
CH25	0.24 ± 0.19	0.14 ± 0.14	0.025*
CH26	0.23 ± 0.19	0.12 ± 0.14	0.027*
CH27	0.19 ± 0.19	0.10 ± 0.12	0.051*
CH28	0.27 ± 0.22	0.14 ± 0.15	0.020*
CH29	0.29 ± 0.16	0.16 ± 0.19	0.014*

CH30	0.28 ± 0.26	0.18 ± 0.15	0.127
CH31	0.19 ± 0.10	0.14 ± 0.15	0.339
CH32	0.23 ± 0.19	0.19 ± 0.16	0.431
CH33	0.26 ± 0.29	0.17 ± 0.20	0.236
CH34	0.29 ± 0.27	0.24 ± 0.20	0.498
CH35	0.31 ± 0.18	0.17 ± 0.15	0.004*
CH36	0.30 ± 0.20	0.11 ± 0.16	<0.001*
CH37	0.33 ± 0.20	0.18 ± 0.17	0.012*
CH38	0.28 ± 0.21	0.11 ± 0.15	0.002*
CH39	0.31 ± 0.14	0.18 ± 0.17	<0.001*
CH40	0.30 ± 0.21	0.20 ± 0.17	0.083
CH41	0.27 ± 0.23	0.25 ± 0.19	0.781
CH42	0.24 ± 0.18	0.17 ± 0.20	0.224
CH43	0.28 ± 0.25	0.20 ± 0.17	0.255
CH44	0.35 ± 0.35	0.28 ± 0.17	0.423
CH45	0.40 ± 0.22	0.22 ± 0.18	0.007*
CH46	0.30 ± 0.17	0.16 ± 0.17	0.012*
CH47	0.32 ± 0.16	0.17 ± 0.20	0.015*
CH48	0.32 ± 0.19	0.16 ± 0.15	0.007*
CH49	0.30 ± 0.16	0.17 ± 0.17	0.016*
CH50	0.31 ± 0.18	0.18 ± 0.19	0.031*
CH51	0.36 ± 0.29	0.34 ± 0.30	0.905
CH52	0.27 ± 0.26	0.19 ± 0.27	0.404

All data are expressed as the mean ± standard deviation

Statistical analysis was conducted using Mann-Whitney *U* test.

* Significant differences between UC and HC group ($p<0.05$).

Table S3. Mean and standard deviation for each channel of HC and CD

	HC	CD	p-value
CH1	0.05 ± 0.15	0.16 ± 0.18	0.057
CH2	0.10 ± 0.18	0.13 ± 0.21	0.772
CH3	0.14 ± 0.13	0.25 ± 0.20	0.074
CH4	0.17 ± 0.16	0.17 ± 0.16	0.950
CH5	0.10 ± 0.17	0.11 ± 0.13	0.773
CH6	0.09 ± 0.12	0.11 ± 0.11	0.608
CH7	0.14 ± 0.12	0.16 ± 0.13	0.497
CH8	0.16 ± 0.13	0.20 ± 0.24	0.510
CH9	0.20 ± 0.13	0.17 ± 0.15	0.611
CH10	0.16 ± 0.16	0.11 ± 0.18	0.419
CH11	0.15 ± 0.19	0.17 ± 0.20	0.864
CH12	0.14 ± 0.22	0.16 ± 0.22	0.787
CH13	0.21 ± 0.17	0.20 ± 0.24	0.942
CH14	0.23 ± 0.19	0.28 ± 0.25	0.486
CH15	0.21 ± 0.25	0.22 ± 0.24	0.961
CH16	0.10 ± 0.18	0.22 ± 0.22	0.090
CH17	0.17 ± 0.16	0.20 ± 0.19	0.705
CH18	0.25 ± 0.17	0.29 ± 0.27	0.566
CH19	0.35 ± 0.21	0.26 ± 0.17	0.148
CH20	0.18 ± 0.20	0.15 ± 0.15	0.597
CH21	0.19 ± 0.07	0.20 ± 0.16	0.724
CH22	0.14 ± 0.17	0.15 ± 0.23	0.891
CH23	0.25 ± 0.15	0.16 ± 0.39	0.484
CH24	0.22 ± 0.11	0.26 ± 0.25	0.475
CH25	0.24 ± 0.19	0.31 ± 0.32	0.380
CH26	0.23 ± 0.19	0.30 ± 0.30	0.413
CH27	0.19 ± 0.19	0.30 ± 0.26	0.114
CH28	0.27 ± 0.22	0.31 ± 0.25	0.592
CH29	0.29 ± 0.16	0.30 ± 0.20	0.898

CH30	0.28 ± 0.26	0.19 ± 0.24	0.327
CH31	0.19 ± 0.10	0.12 ± 0.17	0.255
CH32	0.23 ± 0.19	0.17 ± 0.24	0.412
CH33	0.26 ± 0.29	0.23 ± 0.32	0.805
CH34	0.29 ± 0.27	0.21 ± 0.37	0.513
CH35	0.31 ± 0.18	0.35 ± 0.34	0.670
CH36	0.30 ± 0.20	0.35 ± 0.33	0.537
CH37	0.33 ± 0.20	0.41 ± 0.46	0.604
CH38	0.28 ± 0.21	0.29 ± 0.27	0.864
CH39	0.31 ± 0.14	0.34 ± 0.27	0.693
CH40	0.30 ± 0.21	0.31 ± 0.25	0.990
CH41	0.27 ± 0.23	0.30 ± 0.34	0.774
CH42	0.24 ± 0.18	0.18 ± 0.20	0.327
CH43	0.28 ± 0.25	0.32 ± 0.36	0.696
CH44	0.35 ± 0.35	0.34 ± 0.33	0.903
CH45	0.40 ± 0.22	0.25 ± 0.28	0.093
CH46	0.30 ± 0.17	0.35 ± 0.31	0.520
CH47	0.32 ± 0.16	0.41 ± 0.41	0.460
CH48	0.32 ± 0.19	0.38 ± 0.34	0.567
CH49	0.30 ± 0.16	0.32 ± 0.28	0.808
CH50	0.31 ± 0.18	0.28 ± 0.29	0.731
CH51	0.36 ± 0.29	0.34 ± 0.37	0.891
CH52	0.27 ± 0.26	0.30 ± 0.33	0.741

All data are expressed as the mean ± standard deviation

Statistical analysis was conducted using Mann-Whitney *U* test.

Table S4. Mean and standard deviation for each channel of HC and Active UC

	HC	Active UC	p-value
CH1	0.05 ± 0.15	0.05 ± 0.10	0.985
CH2	0.10 ± 0.18	0.04 ± 0.18	0.526
CH3	0.14 ± 0.13	0.01 ± 0.12	0.034*
CH4	0.17 ± 0.16	0.03 ± 0.04	0.089
CH5	0.10 ± 0.17	0.01 ± 0.04	0.264
CH6	0.09 ± 0.12	0.02 ± 0.04	0.271
CH7	0.14 ± 0.12	0.02 ± 0.08	0.063
CH8	0.16 ± 0.13	0.10 ± 0.14	0.334
CH9	0.20 ± 0.13	0.05 ± 0.08	0.021*
CH10	0.16 ± 0.16	0.07 ± 0.06	0.247
CH11	0.15 ± 0.19	0.07 ± 0.10	0.390
CH12	0.14 ± 0.22	0.16 ± 0.08	0.873
CH13	0.21 ± 0.17	0.11 ± 0.08	0.365
CH14	0.23 ± 0.19	0.06 ± 0.04	0.051
CH15	0.21 ± 0.25	0.04 ± 0.08	0.120
CH16	0.10 ± 0.18	0.10 ± 0.09	0.916
CH17	0.17 ± 0.16	0.06 ± 0.06	0.093
CH18	0.25 ± 0.17	0.12 ± 0.15	0.140
CH19	0.35 ± 0.21	0.18 ± 0.27	0.168
CH20	0.18 ± 0.20	0.15 ± 0.07	0.682
CH21	0.19 ± 0.07	0.11 ± 0.13	0.147
CH22	0.14 ± 0.17	0.12 ± 0.17	0.892
CH23	0.25 ± 0.15	0.27 ± 0.15	0.869
CH24	0.22 ± 0.11	0.09 ± 0.10	0.020*
CH25	0.24 ± 0.19	0.09 ± 0.10	0.069
CH26	0.23 ± 0.19	0.08 ± 0.09	0.091
CH27	0.19 ± 0.19	0.08 ± 0.10	0.205
CH28	0.27 ± 0.22	0.10 ± 0.14	0.105
CH29	0.29 ± 0.16	0.18 ± 0.23	0.236

CH30	0.28 ± 0.26	0.20 ± 0.19	0.554
CH31	0.19 ± 0.10	0.16 ± 0.13	0.592
CH32	0.23 ± 0.19	0.16 ± 0.16	0.432
CH33	0.26 ± 0.29	0.14 ± 0.19	0.368
CH34	0.29 ± 0.27	0.33 ± 0.19	0.795
CH35	0.31 ± 0.18	0.12 ± 0.10	0.022*
CH36	0.30 ± 0.20	0.07 ± 0.13	0.032*
CH37	0.33 ± 0.20	0.16 ± 0.17	0.129
CH38	0.28 ± 0.21	0.06 ± 0.07	0.041*
CH39	0.31 ± 0.14	0.12 ± 0.17	0.020*
CH40	0.30 ± 0.21	0.21 ± 0.19	0.374
CH41	0.27 ± 0.23	0.19 ± 0.15	0.549
CH42	0.24 ± 0.18	0.19 ± 0.15	0.602
CH43	0.28 ± 0.25	0.18 ± 0.14	0.392
CH44	0.35 ± 0.35	0.28 ± 0.21	0.715
CH45	0.40 ± 0.22	0.27 ± 0.20	0.372
CH46	0.30 ± 0.17	0.08 ± 0.13	0.012*
CH47	0.32 ± 0.16	0.12 ± 0.20	0.036*
CH48	0.32 ± 0.19	0.14 ± 0.13	0.052
CH49	0.30 ± 0.16	0.10 ± 0.09	0.009*
CH50	0.31 ± 0.18	0.16 ± 0.15	0.108
CH51	0.36 ± 0.29	0.22 ± 0.23	0.343
CH52	0.27 ± 0.26	0.17 ± 0.18	0.403

All data are expressed as the mean ± standard deviation

Statistical analysis was conducted using Mann-Whitney *U* test.

* Significant differences between Active UC and HC group ($p<0.05$).

Table S5. Mean and standard deviation for each channel of HC and Remission UC

	HC	Remission UC	p-value
CH1	0.05 ± 0.15	0.11 ± 0.14	0.230
CH2	0.10 ± 0.18	0.07 ± 0.16	0.491
CH3	0.14 ± 0.13	0.12 ± 0.16	0.683
CH4	0.17 ± 0.16	0.09 ± 0.12	0.089
CH5	0.10 ± 0.17	0.06 ± 0.08	0.245
CH6	0.09 ± 0.12	0.04 ± 0.11	0.160
CH7	0.14 ± 0.12	0.08 ± 0.14	0.152
CH8	0.16 ± 0.13	0.05 ± 0.14	0.014*
CH9	0.20 ± 0.13	0.06 ± 0.15	0.017*
CH10	0.16 ± 0.16	0.08 ± 0.13	0.099
CH11	0.15 ± 0.19	0.19 ± 0.16	0.605
CH12	0.14 ± 0.22	0.12 ± 0.14	0.772
CH13	0.21 ± 0.17	0.19 ± 0.20	0.755
CH14	0.23 ± 0.19	0.13 ± 0.16	0.068
CH15	0.21 ± 0.25	0.09 ± 0.11	0.027*
CH16	0.10 ± 0.18	0.10 ± 0.12	0.975
CH17	0.17 ± 0.16	0.10 ± 0.13	0.098
CH18	0.25 ± 0.17	0.11 ± 0.19	0.015*
CH19	0.35 ± 0.21	0.11 ± 0.16	<0.001*
CH20	0.18 ± 0.20	0.14 ± 0.13	0.450
CH21	0.19 ± 0.07	0.14 ± 0.15	0.316
CH22	0.14 ± 0.17	0.12 ± 0.16	0.832
CH23	0.25 ± 0.15	0.11 ± 0.18	0.038*
CH24	0.22 ± 0.11	0.20 ± 0.20	0.748
CH25	0.24 ± 0.19	0.15 ± 0.14	0.058
CH26	0.23 ± 0.19	0.12 ± 0.15	0.053
CH27	0.19 ± 0.19	0.11 ± 0.13	0.087
CH28	0.27 ± 0.22	0.15 ± 0.15	0.041*
CH29	0.29 ± 0.16	0.15 ± 0.18	0.012*

CH30	0.28 ± 0.26	0.17 ± 0.15	0.127
CH31	0.19 ± 0.10	0.14 ± 0.16	0.337
CH32	0.23 ± 0.19	0.20 ± 0.16	0.522
CH33	0.26 ± 0.29	0.17 ± 0.22	0.327
CH34	0.29 ± 0.27	0.22 ± 0.21	0.422
CH35	0.31 ± 0.18	0.17 ± 0.16	0.013*
CH36	0.30 ± 0.20	0.12 ± 0.16	0.002*
CH37	0.33 ± 0.20	0.18 ± 0.17	0.020*
CH38	0.28 ± 0.21	0.12 ± 0.16	0.007*
CH39	0.31 ± 0.14	0.19 ± 0.17	0.019*
CH40	0.30 ± 0.21	0.20 ± 0.17	0.092
CH41	0.27 ± 0.23	0.26 ± 0.20	0.898
CH42	0.24 ± 0.18	0.16 ± 0.21	0.227
CH43	0.28 ± 0.25	0.21 ± 0.18	0.339
CH44	0.35 ± 0.35	0.28 ± 0.17	0.452
CH45	0.40 ± 0.22	0.21 ± 0.18	0.007*
CH46	0.30 ± 0.17	0.18 ± 0.18	0.036*
CH47	0.32 ± 0.16	0.18 ± 0.20	0.028*
CH48	0.32 ± 0.19	0.17 ± 0.15	0.016*
CH49	0.30 ± 0.16	0.19 ± 0.18	0.050
CH50	0.31 ± 0.18	0.19 ± 0.20	0.048*
CH51	0.36 ± 0.29	0.40 ± 0.33	0.757
CH52	0.27 ± 0.26	0.20 ± 0.30	0.500

All data are expressed as the mean ± standard deviation

Statistical analysis was conducted using Mann-Whitney *U* test.

* Significant differences between Remission UC and HC group ($p<0.05$).
